# Voice-Related Quality of Life in Patients with Multiple Sclerosis

**DOI:** 10.1155/2012/143813

**Published:** 2012-10-02

**Authors:** Abdul Latif Hamdan, Sahar Farhat, Rami Saadeh, Iyad El-Dahouk, Abla Sibai, Bassem Yamout

**Affiliations:** ^1^Department of Otolaryngology-Head & Neck Surgery, American University of Beirut Medical Center, P.O. Box 11-0236, Beirut, Lebanon; ^2^Department of Internal Medicine, American University of Beirut Medical Center, Beirut, Lebanon; ^3^Epidemiology and Population Health Department, American University of Beirut, Beirut, Lebanon

## Abstract

*Objective*. To investigate the voice-related quality of life in a group of patients with multiple sclerosis. *Participants*. A total of 87 subjects (59 MS subjects and 28 controls) participated in this study. *Main Outcome Measures*. Variables included presence or absence of phonatory symptoms, duration of the disease, the expanded disability status scale (EDSS), the severity of fatigue, and depression. All patients were asked to fill the Voice Handicap Index. *Results*. The average age was 35.47 years + 10.92 with 39% being males. The average duration of the disease was 77.93 months. The EDSS score was 1.94 + 1.84, the FSS score was 4.07 + 2.09, and the HRSD was 7.28 + 7.70. Only 7 subjects out of the 59 had vocal symptoms compared to 3 in the control group. There was no significant difference in the VHI total score between cases (5.9 + 15.5) and controls (5.4 + 8.2). There was a positive correlation between VHI total score, FSS score, and HRSD (*P* values of 0.011 and <0.01. *Conclusion*. The voice-related quality of life in MS is within normal with no disability.

## 1. Introduction

Multiple sclerosis is a neurological disease of the central nervous system characterized by micro- and macroscopic areas of demyelination. The onset is primarily between the ages of 20 and 45 years with a wide spectrum of symptoms and signs [1–3]. There are ubiquitous reports in the literature on the extent of disability as result of being affected by the disease [4–8]. Patients with multiple sclerosis may suffer from visual symptoms, decreased cognitive function, pain, depression, gait ataxia, bladder and sexual dysfunction, and last but not least vocal and speech disorders. All of these symptoms may result in social and physical impairment. Different questionnaires such as the expanded disability status scale for disability status (EDSS), the Hamilton rating scale for depression and fatigue severity scale (FSS) for extent of fatigue have been used to stage the disease and assess the extent of disability and impairment [9–11]. Despite the redundancy in research on the concepts of impairment and disability in MS patients in relation to the various systems in the body, less emphasis has been put on the impact of these disabilities and impairments on quality of life.

Quality of life is defined as “the individual's perceptions of their position in life in the context of culture and value system in which they live, and in relation to their goals, expectations, standards and concerns” as defined by the WHO [12]. Health-related quality of life is conceptualized on those aspects of life quality or function which are influenced by health status. There are several health related quality of life instruments, and these include the generic ones and the disease-specific instruments. The generic measures have been developed without a specific disease in mind whereas the MS-specific health related quality of life is disease specific and includes the Multiple Sclerosis Quality of Life-54, the Disability and Impact Profile, the Multiple sclerosis Impact scale, and the Hamburg Quality of Life Questionnaire in Multiple Sclerosis [5].

Assessment of health related quality of life in patients with MS is important not only in research but also in clinical practice because it allows better decision making in the management and care policy in this group of patients. Several studies have reviewed the impact of multiple sclerosis on health related quality of life in relation to pain symptoms, cognitive function, depression, fatigue, disability, effect of medications, and others [4–8, 13]. Based on an extensive literature review, using the words multiple sclerosis and voice, the authors of this paper could not identify a study that examines voice-related quality of life in patients with multiple sclerosis except for one study by Chiara et al. on the effect of expiratory muscle strength on voice production and voice related quality of life [14]. The study was limited by the small number of subjects with only seventeen participants, 5 of whom withdrew secondary to exacerbation of their expanded disability status scale. Assessment of voice related quality of life not only allows new therapeutic interventions but also may result in a change in the care of policy of affected patients. 

The purpose of this study is to investigate the voice related quality of life in a large group of patients with multiple sclerosis. The Voice Handicap Index, one of many patient-reported outcome measures that have gained popularity in both the clinical and research milieus, will be used in this investigation. 

## 2. Materials and Method

### 2.1. Ethical Considerations

A total of 87 subjects were asked to participate in this study after having read and signed the informed consent approved by the Institutional Review Board.

Confidentiality was maintained throughout the data collection process. All data was kept under lock and key and it was accessible only to the principle investigator and his research team. 

### 2.2. Participants

Subjects with recent history of upper respiratory tract infection, laryngeal manipulation, or previous laryngeal surgery were excluded from this study. The subjects were divided into two groups, one with MS consisting of 59 subjects and one as control consisting of 28 healthy subjects matched according to age and gender. Patients with MS were referred from the private clinic of the neurology service. 

### 2.3. Tools

Demographic variables included age, gender, and history of smoking. Other variables taken included presence or absence of phonatory symptoms, duration of the disease in months, the expanded disability status scale (EDSS) reflecting the severity of the disease, the severity of fatigue, and depression. The type of the disease was classified according to the progression of the disease as relapsing remitting (RRMS), secondary progressive (SPMS), relapsing/progressive (RPMS), and primary progressive (PPMS). For the disability status, a score between 0 and 3 was considered mild, between 3.5 and 6 as moderate, and above 6 as severe [9]. The presence and severity of fatigue were assessed by means of the fatigue severity scale (FSS). This test consists of nine items concerning fatigue each rated on a 7-point scale. The average rating is computed for all nine items with higher scores indicating increasing fatigue. A cut-off point of 4 was taken in this study. This test was chosen in view of its accepted consistency, stability, and sensitivity to clinical stage [10]. Depression was evaluated using the Hamilton Rating Scale for Depression (HRSD) which consists of 21 items rated from 0 to 4 with a higher score indicating increasing severity of depression [11]. It is worth noting that a cut-off of 10 (>10) was used to define the prevalence of depression.

All patients were asked to fill the Voice Handicap Index. It is a patient-based self-assessment tool that consists of 30 items distributed over three domains: functional, physical, and emotional. The functional subscale describes the “impact of voice disorders on daily activities,” the physical subscale describes patients' self-perceptions of laryngeal discomfort or the voice output characteristics, and the emotional subscale illustrates patients, “affective response to voice disorders”. The VHI total score ranges between 0 and 120 a high number indicates greater severity of voice problem [15]. It is worth noting that a cut-off of 15 is usually used to identify individuals with voice problems [16].

Demographic characteristics of the participants were examined separately for cases and controls using frequency distributions for categorical variables (gender, smoking and type of MS) and means with standard deviations for continuous ones (age, duration of MS, EDSS, FSS score, and HRSD).

The differences between cases and controls in terms of VHI total score and subscores (VHI P, VHI F, and VHI E) were calculated using independent samples *t*-tests. Bivariate Spearman's correlation was used to measure the association between VHI score and sub scores with FSS score, HRSD, EDSS score, and disease duration among cases and with FSS score and HRSD among controls. A *P*  value < 0.05 was considered as significant. Analyses were performed using Statistical Analysis Package for Social Sciences (SPSS, version 19.0 Chicago, IL, USA).

## 3. Results

### 3.1. Demographic Data

A total of 59 patients with MS were enrolled in this study. The average age was 35.47 years + 10.92 with 39% being males. Close to 90% were in the RRMS stage of the disease, and the average duration of the disease was 77.93 months. The EDSS score was 1.94 + 1.84, the FSS score was 4.07 + 2.09, and the HRSD was 7.28 + 7.70. It is worth noting that 52.5% of patients and 3.57% of controls had fatigue based on an FSS cut-off score of 4. Similarly, 28.8% of patients and 3.57% of controls had depression based on an HRSD cut-off of 10. Only 7 subjects out of the 59 had vocal symptoms compared to 3 in the control group. The symptoms were mainly vocal breaks in three, vocal break and fatigue in one, and nonspecific dysphonia in the remaining. See Table 1.

### 3.2. Voice Handicap Index Score for Patients with Multiple Sclerosis and Controls

The mean score for the VHI in the diseased group was 5.9 + 15.5, whereas for the control the mean it was 5.4 + 8.2. There was no significant difference in the VHI total score between cases and controls. The scores for the physical, functional, and emotional components were 2.4 + 5.4, 2.2 + 5.4, and 1.4 + 5.6, respectively, with no significant difference compared to the corresponding scores of the control group. See Table 2. It is worth noting that despite the lack of significant difference in the mean score among the two groups (patients and controls) the percentage of patients with VHI score above 15 (>15) was 8.47% compared to 3.57% in the control group. The mean score of VHI in patients with vocal symptoms was 29.7 versus 2.7 in those with no vocal symptoms.

### 3.3. Association between the VHI Scores and EDSS, FSS, HRSD, and Duration of Disease in Cases and Controls

Among cases, there was a positive correlation between VHI total score and FSS score and HRSD (*P* values of 0.011 and <0.001). Similarly there was a positive correlation between the three components of the VHI, namely, physical, emotional, and functional, and the FSS score and HRSD as indicated in Table 3. It is worth noting that 74% of patients with vocal symptoms had fatigue and depression compared to 50% and 23.7%, respectively, in patients with no vocal symptoms. Among the controls, the associations were overall weak and not significant as indicated in Table 4.

## 4. Discussion

The voice is a sensitive neurophysiological signal that reflects the status of the nervous system and its pathways. It is well recognized that MS is a condition that can negatively affect voice and health related quality of life in various domains [17–20]. These comprise the physical and functional disabilities, psychological status and well-being, economic status, social interactions, and last but not least the religious and spiritual status [21]. 

### 4.1. Comparison with Other Studies

Understanding the quality of life in relation to specific diseases and systems can be used as a predictive factor for the development of any disease and for the advancement in its treatment. There are several reports in the literature investigating the association between MS disabilities and impairment in relation to quality of life. In Rudick et al.'s report on the relationship between disease activity and health related quality of life in relapsing multiple sclerosis, the mean baseline SF-36 scores were significantly less than the general population and correlated with Expanded Disability Status Scale scores, sustained disability progression, relapse number, and increased volume of brain magnetic resonance imaging lesions [13]. In another study on 103 patients with MS, using the Multiple Sclerosis Quality of Life-54, there was a moderate inverse relationship between disability level and the MSQOL-54 physical composite score and a moderate-to-strong inverse correlation between depression or fatigue severity and both the physical and mental composite scores. More so, depression, fatigue, and disability levels were confirmed to be significant and independent predictors of quality of life [4]. In a longitudinal evaluation of cognitive functions in MS patients over three years, using several tests such as the SF-36 for quality of life and the Luria Frontal Lobe Syndrome test, the results indicated that the frontal component of cognitive functions and behavioural memory involvement were related to worsening of QOL in particular in the physical functioning and the mental health of SF-36 [7]. Nortvedt et al. have estimated the relationship between the presence and degree of sexual disturbance/bladder dysfunction and the patient's quality of life using the SF-36 Health Survey. The results indicated that patients with sexual disturbances had markedly reduced scores on all eight SF-36 scales and scored 1.5  SD lower than a normal population on the social functioning scale. The conclusion was that bladder and sexual dysfunctions are associated with a marked reduction in quality of life in patients with MS [8].

Although commonly masked by speech disorder, vocal symptoms such as vocal fatigue, voice breaks, and phonatory instability still prevail in multiple sclerosis with little knowledge of their impact on life. The prevalence of phonatory symptoms varies in various reports and one-third of patients with MS can have varying degree of dysphonia compared to 7.4% of controls. Other studies have reported a prevalence rate of 70% versus 33% of controls [17–20]. The authors of this paper have reported the most common symptoms to be vocal breaks and vocal fatigue in 10 out of 40 patients with MS versus zero prevalence of voice breaks in the control group [20]. In a study by Konstantopoulos et al. on the phonatory instability in patients with MS, the results indicated that most of the variables used, namely, the mean fundamental frequency, standard deviation and jitter, differentiated the MS group from the controls [18]. The results of our investigation indicate that around 12% of patients with MS have reported voice problems, a relatively low percentage compared to the literature. This could be attributed to the methodology used, namely, self-reported collection of data, and second to the apprehensive approach of many patients in pointing additional symptoms as this might indicate or allude to a worse prognosis. Another important point to note in the understanding of this given low percentage of phonatory symptoms is that the MS subject population of this study represents only a part of the MS spectrum given the fact the mean duration of disease was almost 6 years and the EDSS score was less than 3.

Despite the fact that vocal dysfunction is often reported in patients with multiple sclerosis, the voice related quality of life in MS patients remains unknown. The authors of this paper have chosen the VHI, one of the strongest psychometric measures, as a patient-reported outcome measure of voice related quality of life [22]. This tool has been developed by Jacobson and colleagues in 1998 in order to measure self-perception of voice problems, through the evaluation of the emotional, functional, and physical aspects of voice. It has been translated into several languages including German, Taiwanese, Spanish, and Portuguese, and has been evaluated for reliability and validity. Since then, it has been used in clinical research to measure the various aspects of voice disorders in both professional and nonprofessional voice users and to assess pre- and post-treatment outcomes [23]. In our study the questionnaire was filled by all the subjects with MS with the purpose of assessing whether voice related quality of life is being any different in diseased patients versus controls. 

### 4.2. Study Findings

The results of our study revealed that the mean score of the VHI total was less than 20 meaning that voice related quality of life is normal with no major impact. The results further indicate that there is no significant difference in the mean score of VHI in MS patients and controls. Our results are not in accordance with those of Chiari et al. In their study on 17 participants with MS, the effect of expiratory muscle strength training on maximal expiratory pressure and voice was investigated [14]. The voice related quality of life score the subscale scores and as well for both social and physical components were significantly lower in patients with MS versus controls. However, expiratory muscle training did not have an effect on the quality of life of voice-related issue. The results of this study were limited by the small number of subjects and the lack of insight on the impact of dysphonia on the emotional domain of quality of life. 

The low VHI scores in our diseased patients could be attributed to several factors. First is the little disability in our diseased group as indicated by the low EDSS score below 3 reflecting mild if any disability, even though one can argue that the prevalence of phonatory symptoms is not associated with the EDSS score as previously reported by the authors of this paper 19. Second is the stage of the disease as most of our patients, close to 90%, were in the relapsing/remitting stage, and last but not least is the low fatigue severity score 4.02, further indicating the relatively minimal fatigability experienced by the MS patients.

What is important to note in our investigation is the correlation between the total VHI score and sub scores with the FSS and HRSD score among cases, as indicated, for instance that the FSS score (fatigue) explains 33% of the VHI score and HRSD (depression) explains a 50%. This correlation is easy to understand given that voice is a reflection of the entire being and thus infers the overall physical and emotional status of the individual. With respect to the association with the fatigability severity scale (*P*  value  0.010), this concurs the fact that the overall fatigability score is intimately related to the phonatory symptoms in MS patients as most reports indicate that vocal fatigue and vocal breaks are the predominant symptoms in affected individuals [17–21]. This was substantiated in our investigation where the main phonatory symptoms in our MS subjects were, namely, fatigue and breaks. According to several investigations, vocal fatigue is explained on the basis of several theories related to the extent of hydration, rheologic properties of the vocal folds, and most importantly to the respiratory fatigue [24]. Respiratory fatigue and poor breathing support can affect phonation and result in vocal functional disorders with an array of symptoms including vocal fatigue. Smeltzer et al. have demonstrated that respiratory muscle weakness, though subclinical, is present in MS patients who experience throat clearing and cough [25]. Based on the aforementioned reports, one can conclude the association between the VHI scores and the FSS score. 

With respect to the association between VHI scores and depression, several studies have confirmed the correlation between mental status and voice. In a study by Park CK et al., mood status of young subjects could be estimated using acoustic parameters [26]. Similarly in a prospective study on 119 subjects looking at the relationship between depression and voice using the VHI questionnaire and the Beck Depression Inventory-Fast Screen (BDI-FS) survey, the results indicated the presence of a correlation between VHI emotional domain scores and BDI-FS scores [27]. 

This study carries two limitations. First is the lack of an objective outcome measure for voice such as acoustic analysis, and second is the lack of laryngeal examination. However it is worth noting that the purpose of this investigation was to measure the voice related quality of life as perceived by the patient and not the physician. For that purpose, the Voice Handicap Index was used in all subjects with MS with no input from the treating physician. Our results indicate that vocal symptoms, when reported by the patient, are not that common and have little impact if any on the quality of life. 

## 5. Conclusion

The prevalence of dysphonia in patients with MS is low when patient-reported outcome measures such as the VHI are used. The voice related quality of life in MS is within normal with no disability.

## Figures and Tables

**Table 1:** Demographics.

Variables	Cases *N* = 59		Control *N* = 28	
	Mean	SD	Mean	SD
Age	35.47	10.92	33.25	11.47
Duration of MS (months)	77.93	79.82	N/A	N/A
EDSS	1.94	1.84	N/A	N/A
FSS score	4.07	2.09	2.22	0.94
HRSD	7.28	7.70	3.18	3.12

	*N*	%	*n*	%

Gender (% male)	23	39.0	11	39.3
Smoking (% yes)	24	40.7	8	28.6
Type of MS				
RRMS	52	89.7	N/A	N/A
SPMS	6	10.3	N/A	N/A
Phonatory symptoms	7	11.9	3	10.7

*Some numbers do not add up to the total because of missing values.

**Table 2:** Difference between cases and control in terms of VHI scores.

Variables	Cases		Control		*P* value
	Mean	±SD	Mean	±SD
VHI total	5.92	15.47	5.32	8.21	0.849
VHI P	2.36	5.36	1.86	2.92	0.647
VHI F	2.15	5.37	2.18	3.48	0.981
VHI E	1.41	5.60	1.29	2.80	0.914

**Table 3:** Association of VHI scores with selected variables among cases.

Variables	VHI total		VHI P		VHI F		VHI E	
	Correlation coefficient (*r*)	*P* value	Correlation coefficient (*r*)	*P* value	Correlation coefficient (*r*)	*P* value	Correlation coefficient (*r*)	*P* value
FSS score	0.331	0.011	0.349	0.007	0.319	0.014	0.273	0.036
HRSD	0.505	<0.001	0.554	<0.001	0.478	<0.001	0.407	0.001
EDSS score	−0.035	0.803	0.030	0.832	−0.053	0.703	−0.073	0.599
Disease duration	0.093	0.486	0.163	0.222	0.059	0.660	0.045	0.736

**Table 4:** Association of VHI scores with selected variables among controls.

Variables	VHI total		VHI P		VHI F		VHI E	
	Correlation coefficient (*r*)	*P* value	Correlation coefficient (*r*)	*P* value	Correlation coefficient (*r*)	*P* value	Correlation coefficient (*r*)	*P* value
FSS score	−0.229	0.240	−0.291	0.132	−0.171	0.385	−0.176	0.370
HRSD	−0.072	0.716	0.005	0.979	−0.077	0.698	−0.109	0.580
